# Sensory over-responsivity: parent report, direct assessment measures, and neural architecture

**DOI:** 10.1186/s13229-019-0255-7

**Published:** 2019-02-04

**Authors:** Teresa Tavassoli, Anne Brandes-Aitken, Robyn Chu, Lisa Porter, Sarah Schoen, Lucy Jane Miller, Molly Rae Gerdes, Julia Owen, Pratik Mukherjee, Elysa J. Marco

**Affiliations:** 1 Department of Psychology and Clinical Language Sciences, University of Reading, Reading, UK; 2 Department of Applied Psychology, New York University, New York, NY USA; 3 Sensory Therapies and Research (STAR) Institute, Greenwood Village, CO USA; 4 University of Colorado Denver, Denver, CO USA; 5 Rocky Mountain University of Health Professionals, Provo, UT USA; 6 Department of Neurology, University of California San Francisco, San Francisco, CA USA; 7 Department of Radiology, University of Washington, Seattle, WA USA; 8 Department of Radiology and Biomedical Imaging, University of California San Francisco, San Francisco, CA USA; 9 Department of Pediatrics, University of California San Francisco, San Francisco, CA USA; 10 Research Division, Cortica Healthcare, San Rafael, CA USA

**Keywords:** Sensory over-responsivity, Diffusion Tensor Imaging, Assessment, Neurodevelopmental disorder, autism, Sensory processing disorder

## Abstract

**Background:**

Sensory processing difficulties are common across neurodevelopmental disorders. Thus, reliable measures are needed to understand the biological underpinnings of these differences. This study aimed to define a scoring methodology specific to auditory (AOR) and tactile (TOR) over-responsivity. Second, in a pilot cohort using MRI Diffusion Tensor Imaging, we performed a proof of concept study of whether children with AOR showed measurable differences in their white matter integrity.

**Methods:**

This study included children with AOR and TOR from a mixed neurodevelopmental disorder cohort including autism and sensory processing dysfunction (*n* = 176) as well as neurotypical children (*n* = 128). We established cohorts based on sensory over-responsivity using parent report (Short Sensory Profile (SSP)) and direct assessment (Sensory Processing-Three Dimensions: Assessment (SP-3D:A)) measures. With a subset of the children (*n* = 39), group comparisons, based on AOR phenotype, were conducted comparing the white matter fractional anisotropy in 23 regions of interest.

**Results:**

Using direct assessment, 31% of the children with neurodevelopmental disorders had AOR and 27% had TOR. The inter-test agreement between SSP and SP-3D:A for AOR was 65% and TOR was 50%. Children with AOR had three white matter tracts showing decreased fractional anisotropy relative to children without AOR.

**Conclusions:**

This study identified cut-off scores for AOR and TOR using the SSP parent report and SP-3D:A observation. A combination of questionnaire and direct observation measures should be used in clinical and research settings. The SSP parent report and SP-3D:A direct observation ratings overlapped moderately for sensory related behaviors. Based on these preliminary structural neuroimaging results, we suggest a putative neural network may contribute to AOR.

**Electronic supplementary material:**

The online version of this article (10.1186/s13229-019-0255-7) contains supplementary material, which is available to authorized users.

## Background

Sensory processing dysfunction (SPD), manifests as difficulty interpreting the sensory world in an adaptive way, is common across children with neurodevelopmental disorders (NDD), including children who meet the categorical label of autism spectrum disorder (ASD) [1, 2]. Under the umbrella of SPD, there are three suggested primary subtypes: difficulties modulating sensory input, difficulties discriminating sensory information, and difficulties with sensory-based motor control [3]. While these challenges can exist independently, they often co-occur. The Diagnostic and Statistical Manual-5 (DSM-5) now includes hyper- and hypo-reactivity to sensory input (characteristic of sensory modulation) as a core criteria for ASD, which has prompted additional interest and focus on sensory modulation [4].

Previous research suggests that one aspect of sensory modulation, sensory over-responsivity (SOR), occurs most frequently in the auditory and tactile domains; thus, these sensory domains are the focus of this investigation [5]. We chose to focus on SOR given the distress associated with it [6]. Over-responsivity manifests as extreme adverse or avoidant responses to sensory stimulation, such as covering ears and running from the room in response to a vacuum cleaner, blender, or automatic flushing toilet (auditory over-responsivity (AOR)). In the tactile domain, sensory over-responsivity modulation difficulties may manifest as refusal to wear clothing (particularly underwear), not liking to be touched, and not wanting to touch certain materials, leading to significant household disruption and social challenges (tactile over-responsivity (TOR)).

We seek to investigate the structural underpinnings of SOR to determine if there is a unique, architectural neural signature that can be used as a biomarker for intervention. This study focuses on auditory over-responsivity (AOR) and tactile over-responsivity (TOR) in a broad neurodevelopmental cohort, taking a Research Domain Criteria (RDoC)-inspired “sensory-first” approach [7]. The goal is to compare direct assessment and parent report measures of AOR and TOR in a pediatric cohort and to explore the neural architecture of SOR in children across categorical diagnoses.

### Characterizing sensory over-responsivity in children with neurodevelopmental disorders

Sensory responsivity measures include parent reports, expert observation, and psychophysiological testing [8–11]. Currently, parent report measures often assess sensory modulation but include a combination of modulation phenotypes as well as other aspects of sensory processing [12–18]. The Sensory Processing-Three Dimensions: Inventory quantifies sensory domains (vision, hearing, touch, and movement) by modulation and discrimination, as well as sensory-based motor challenges [19, 20]. The Sensory Sensitivity Questionnaire and the Sensory Experiences Questionnaire characterize sensory modulation specifically for children with ASD [21, 22]. The Sensory Profile (SP) has been validated cross-culturally and across clinical cohorts using sensory quadrant and section scoring methodology [15, 23–29]. The Short Sensory Profile (SSP), derived from the SP, has been used to differentiate typically developing children from children with ASD [8, 16, 18, 30, 31]. The SSP and other parent reports have made significant contributions to the research and clinical understanding of sensory dysfunction and have been instrumental for “trait-based” assessment. Although important for describing trait behavior, caregiver reports are subjective by nature and often affected by previous experience and expectation. Consequently, while they are a critical component of a thorough and appropriate clinical formulation, they are less ideal for objective state assessment and in previous work have shown less correlation with brain structure than direct assessment [32]. Furthermore, a recent factor analysis in children with autism spectrum disorder questions the research validity of the SSP’s current factor structure, suggesting that two questions in particular are specific to AOR, which is one of the two sensory domains highlighted in this current project [33]. With a more limited but specific SOR subset of questions from the SSP, we aim to better assess the phenotype between auditory and tactile over-responsivity in this cohort of children with and without neurodevelopmental challenges.

We suggest that often parent report measures, the totals and even some of the current subscales, coalesce a more complex cluster of behavioral observations while direct assessment, such as the one included in this study, aims to assess a single sensory domain at a single point in time, in a controlled environment, on a singular processing ability (sensory over-responsivity). Several sensory observation measures exist for young children such as the Sensory Integration and Praxis Tests (SIPT), the Sensory Processing Assessment for Young Children (SPA), the Tactile Defensiveness and Discrimination Test-Revised (TDDT-R), and the Infant Test of Sensory Functioning [34–38]. A previous study using the Sensory Processing-Three Dimensions: Assessment (SP-3D:A), a direct sensory modulation observation for individuals 3 to 21 years of age, identified the most differentiating items for children, adolescents, and young adults with autism [5]. Moreover, previous work investigated reliability and validity but cut-off scores to enable categorization for clinical utility and direct research group comparison have not yet been developed [39, 40]. Hence, this study seeks to advance the field of sensory assessments by comparing the auditory and tactile over-responsive items for children with neurodevelopmental disorders using parent report (SSP) and direct assessment (SP-3D:A) and by providing cut-off scores. While other observational measures focus on one sensory domain, the SP-3D:A is ideally suited for this task, as it includes characterizations of SOR in both auditory and tactile domains [9].

### Neural architecture of sensory processing to date

The neural architecture, both structural and functional, of sensory processing in individuals with autism has been explored using a variety of techniques and paradigms including EEG, MEG, fMRI, MRS, and DTI [41–46]. This study focuses on refining our understanding of the structural differences underlying auditory and tactile over-responsivity using DTI across neurodevelopmental conditions. Prior DTI work has characterized the neural underpinnings of sensory processing differences more broadly in children with ASD and SPD but has not taken a more parsimonious approach [32, 45, 47]. For example, Chang et al. reported robust alterations of posterior white matter microstructure in children with broadly defined SPD relative to typically developing children (TDC) [32]. This investigation found strong correlations between fractional anisotropy (FA), a measure of microstructural integrity, and parent report and direct assessment measures of tactile and auditory discrimination across all children. However, direct assessment of sensory discrimination showed stronger and more continuous mapping to underlying white matter integrity than the parent report measures. Additionally, in children with ASD, Pryweller et al. reported decreased FA in the inferior longitudinal fasciculus (ILF), which correlated directly with measures of TOR (defensiveness), suggesting atypical connectivity between the limbic system and multisensory integration regions [46]. This finding offers a preliminary explanation for the dysregulated emotional valence applied to non-noxious tactile stimuli. While the current literature has provided initial evidence for structural correlates of sensory processing dysregulation, further research is needed to specify the existence of neural tracts associated with specific domains of sensory over-responsivity. This approach will contribute to developing novel, targeted interventions aimed at atypical structural connectivity in children with neurodevelopmental disorders. By assessing connectivity before and after trainings targeting over-responsivity, we hope to be able to determine whether applied interventions are indeed leading to measurable change. But first, we need to know where to look and what to measure. This study is an initial foray in this next step. In this study, we hypothesize that direct assessment of AOR and TOR will show strong inter-test agreement with corresponding parental report behaviors in a NDD cohort and that sensory-first categorization using direct assessment of AOR will identify a more succinct subset of white matter tracts than previously identified using parent report.

## Methods

### Demographics

#### Experiment 1: direct auditory and tactile over-responsive phenotyping

A total of 304 participants were enrolled in experiment 1—128 typically developing children (TDC) and 176 children with NDD (see Table 1). The NDD group was composed of 100 children with SPD (55 female, age 8.5 ±  3.0 years) and 76 children with ASD (10 female, age 9.6 ±  3.0 years). ASD cohort inclusion included a community diagnosis of ASD, a score of ≥ 15 on the Social Communication Questionnaire (SCQ) and/or a score of ≥ 25 on the autism quotient (AQ), and a confirmed ASD classification with the Autism Diagnostic Observation Schedule, Second Edition (ADOS-2) [48–50]. Participants in the SPD and TDC groups scored below cut-off criteria on the AQ or SCQ. Participants in the SPD cohort had an SPD designation from a community occupational therapist and/or a score in the “definite difference” range (< 2% probability) in one or more of the SP section scores.

**Table 1:** Participant demographics—experiment 1

	TDC *n* = 128	NDD *n* = 176	*p* value
Age (years)	9.8 ± 2.9	8.9 ± 3.1	*F* = 6.02, *p* = .01
Gender (m/f)	66/62	111/65	*F*= 3.28, *p* = .07
FSIQ*	111.9 ± 18.1	101.4 ± 16.3	*F* = 13.4, *p* < .001

*TDC* typically developing children, *NDD* neurodevelopmental disorders, *FSIQ* Full Scale IQ

*FSIQ available for UCSF and Seaver Autism Center cohorts only (TDC *n* = 66, NDD *n* = 76)

Participants in this Sensory Processing Disorder Consortium project were recruited from the University of California, San Francisco (UCSF) Sensory Neurodevelopment and Autism Program, the STAR Institute in Denver, Colorado, and the Icahn School of Medicine at Mount Sinai in New York (Seaver Autism Center). All parents provided written consent on behalf of their children, while children provided informed assent in accordance with each site’s institutional review board. Given the retrospective nature of this study, not all children were administered all measures. All typically developing children in this collaborative cohort who had the specified assessment were included for establishing cut-off scores; children who had both direct assessment using the SP-3D:A and parent report using SSP were included in the phenotype comparison (*n* = 235). Children from the UCSF site received the Wechsler Intelligence Scale for Children-Fourth Edition to evaluate cognition. Children from the Seaver Autism Center received the Wechsler Abbreviated Scale of Intelligence [51, 52].

#### Experiment 2: structural neural assessment of auditory over-responsivity

For structural Diffusion Tensor Imaging (DTI) analysis, we included 39 boys from UCSF who successfully completed direct sensory assessment and neuroimaging assessment (ASD, *n* = 13 (mean age 11 ± 2 years); SPD, *n* = 8 (mean age 11 ± 1 year); and TDC, *n* = 18 (mean age 12 ± 1 year)) (see Table 2). Fifteen children scored above cut-off for AOR. This cohort has been previously described in Chang et al. [32]. Due to a small sample size in the TOR cohort, only eight children met TOR cut-off and we constrained the DTI analysis to the auditory domain.

**Table 2:** Participant demographics—experiment 2

	AOR *n* = 15	NO-AOR *n* = 24	*p* value
Age (years)	11.33 ± 1.11	11.74 ± 1.48	*p* = .37
VCI	110.0 ± 19.43	113.7 ± 16.14	*p* = .53
PRI	109.2 ± 14.62	112.8 ± 12.23	*p* = .35

*VCI* verbal comprehension index, *PRI* perceptual reasoning index, *AOR* above auditory over-responsive cut score, *NO-AOR* below auditory over-responsive cut score

## Measures

### Sensory phenotyping measures

#### Parent report: Short Sensory Profile Questionnaire

The SSP includes 38 items in which parents rate how often their child shows a particular sensory behavior using a five-point Likert scale ranging from always (1) to never (5). Higher scores reflect more sensory-typical behavior. To align with the SP-3D:A, we inverted the scoring with never (1) and always (5). Thus, higher scores on both parent report and direct assessment will reflect greater SOR. The SSP has high internal reliability (.90–.95) and shows sensory differences in up to 90% of children and adults with ASD compared to controls [8, 30]. To achieve an SOR-specific score for the auditory and tactile domains, we chose items reflecting SOR behaviors by clinical consensus (TT, EJM, SS, LJM, RC, LP) (see Table 3). We included items that represent clear signs for SOR, rather than items that could be explained by other factors such as attention difficulties (e.g., we excluded auditory filtering items such as “Can’t work with background noise”).

**Table 3:** Short Sensory Profile items for tactile and auditory over-responsivity

SSP:TOR	SSP:AOR
1. Expresses distress during grooming	34. Responds negatively to unexpected or loud noises
2. Avoids going barefoot, especially in sand or grass	35. Holds hands over ears to protect ears from sound
3. Reacts emotionally or aggressively to touch 4. Withdraws from splashing water	
5. Has difficulty standing in line or close to other people 6. Rubs or scratches out a spot that has been touched	

*SOR* sensory over-responsivity, *SSP* Short Sensory Profile, *TOR* tactile over-responsivity, *AOR* auditory over-responsivity

#### Clinician-administered assessment: Sensory Processing-Three Dimensions: Assessment

The SP-3D:A, a structured observational tool measuring behavioral response to specific sensory stimuli, includes probes that are administered by a STAR Institute-trained, research-reliable experimenter. The internal reliability is high (alpha = .94) [9]. Here, we included three auditory probes: “Find a picture,” during which participants cross out symbols with loud background noise; “Orchestra time,” in which participants play along with loud music using provided instruments; and “Sound and pictures,” where participants identify sounds such as a vacuum cleaner or dog barking. The tactile probes included the following: “Paint your arm,” during which participants paint their arm with a feather, a brush, and a rough sponge; “Goo,” in which participants remove two plastic animals from goo; and “Fishing,” requiring participants to retrieve plastic fish from a bucket of ice water. The following SOR behaviors during the game are given a score of 0 (not present) or 1 (observed): adverse response (0/1) (e.g., startling during sounds, grimacing), discomfort, worries, and/or avoidance (0/1) (e.g., stating worries about the task, refusing to do it). For auditory over-responsive (SP-3D:AOR) and tactile over-responsive (SP-3D:TOR) composite scores, we summated the SOR behavior scores for the three games. Behaviors observed during, not prior to, or between tasks are included. Thus, each composite, SP-3D:AOR and SP-3D:TOR, ranges from 0 to 6. A child who does not show any OR behaviors would score a 0, and a child who scores for adverse response (1) and avoidance (1) on all three selected games would score a 6.

### DTI acquisition

MR imaging was performed on a 3 T Tim Trio scanner (Siemens, Erlangen, Germany) using a 12-channel head coil with an axial 3D magnetization-prepared rapid acquisition gradient-echo T1-weighted sequence (TE = 2.98 ms, TR = 2300 ms, TI = 900 ms, flip angle of 90°) with in-plane resolution of 1 × 1 mm on a 256 × 256 matrix and 160 1.0-mm contiguous partitions. Whole-brain diffusion imaging was performed with a multislice 2D single-shot twice-refocused spin echo-planar sequence with 64 diffusion-encoding directions, diffusion-weighting strength of *b* = 2000 s/mm^2^, iPAT reduction factor of 2, TE/TR = 109/8000 ms, NEX = 1, interleaved 2.2-mm-thick axial slices with no gap, and in-plane resolution of 2.2 × 2.2 mm on a 100 × 100 matrix. An additional image volume was acquired with no diffusion weighting (*b* = 0 s/mm^2^). The total diffusion acquisition time was 8.7 min. Structural MRI for all children was reviewed by Dr. Pratik Mukherjee, a pediatric neuroradiologist, blind to cohort assignment. No clinically significant structural anomalies were identified.

### DTI pre-processing

The diffusion-weighted images were corrected for motion and eddy currents using Functional Magnetic Resonance Imaging of the Brain Software Library’s Linear Image Registration Tool (FSL; FLIRT1) with 12-parameter linear image registration [53]. All diffusion-weighted volumes were registered to the reference *b* = 0 s/mm^2^ volume. To evaluate participant movement, we calculated a scalar parameter quantifying the transformation of each diffusion volume to the reference. As reported in previous studies, 16 children were excluded for DTI artifacts and/or median relative displacement between volumes greater than 2 mm, where a volume represents a single diffusion directional measurement of the entire brain. This left a total of 39 children with DTI datasets meeting quality control criteria and direct assessment with the SP-3D:A. A heteroscedastic two-sample Student’s *t* test verified that there were no significant differences between these AOR and TDC groups in movement during the DTI scan (*p* > 0.05). The non-brain tissue was removed using the Brain Extraction Tool. FA was calculated using FSL’s DTIFIT at every voxel, yielding FA maps for each participant.

### Region of interest DTI analysis

Tract-Based Spatial Statistics in FSL was used to skeletonize and register the diffusion maps for each participant in order to perform voxel-wise comparisons along the white matter skeleton [54]. First, each participant’s FA map was non-linearly registered to each other participant’s FA map to identify the most representative FA map as a registration target. The registered maps were then averaged and skeletonized to the center of the white matter. Next, each participant’s FA data was projected onto this mean skeleton to obtain skeletonized FA maps per participant. Tract regions of interest (ROIs) were created according to The Johns Hopkins University ICBM-DTI-81 White-Matter Labeled Atlas [55]. Right and left hemisphere ROI tracts were highly correlated (*r* ≥ .50, *p* ≤ .001); thus, an average diffusion value across right and left tracts was created for each participant.

## Statistical analysis

### Experiment 1: cut score analysis and inter-test reliability

SPSS 24 was used to analyze the SSP and SP-3D:A data. Cut scores were designated at one standard deviation above the TDC group’s mean (rounded to the nearest whole integer) similar to the development of the Sensory Experience Questionnaire cut-off scores [22]. Inter-rater reliability was calculated by measuring the absolute agreement between SSP:AOR and SP-3D:AOR and between SSP:TOR and SP-3D:TOR. Chi-square analysis was used to assess differences in over-responsivity between the NDD and TDC group.

### Experiment 2: DTI analysis between children with and without auditory over-responsivity

Utilizing the SP-3D:AOR cut score determined in experiment 1, we categorized the neuroimaging cohort to either an AOR (*n* = 15) or NO-AOR (*n* = 24) cohort. Due to a small sample size in the tactile domain (*n* = 8), we focused on AOR for experiment 2. We analyzed mean FA differences in 22 bilateral ROIs. We constructed ANOVAs using the categorical predictor variable for AOR (two levels: above or below cut score), and the outcome variables were the 22 ROIs. We review these findings both with and without false detection rate (FDR) correction to *p* values (0.05) for each ANOVA test.

## Results

### Experiment 1

Cohort groupings based on the TDC results of parent report and direct assessment measures were determined (see Table 4 and Additional file 1: Figure S1, Additional file 2: Figure S2, and Additional file 3: Figure S3). Specifically, for each measure, we calculated the TDC mean + 1 SD. We then, per mathematical convention, rounded to the nearest whole integer (i.e., 1.3 would round down to 1 and 9.7 would round up to 10). The resulting number was used as the dividing line between SOR and NO-SOR groups such that children who scored greater than the integer were placed in the SOR group while those scoring less than or equal to the value were included in the NO-SOR group.

**Table 4:** Cohort assignment for auditory and tactile over-responsivity

	TDC mean ± SD (*n*)	+ 1 SD	NO-SOR	SOR
SP-3D:A—auditory	.38 ± .98 (127)	1.3	0–1	≥ 2
SP-3D:A—tactile	.29 ± .72 (128)	1.0	0–1	≥ 2
SSP—auditory	3.0 ± 1.4 (89)	4.4	2–4	≥ 5
SSP—tactile	7.5 ± 2.2 (92)	9.7	6–10	≥ 11

*TDC* typically developing children, *SOR* sensory over-responsivity, *SP-3D:A* Sensory Processing-Three Dimensions: Assessment, *SSP* Short Sensory Profile

Using direct assessment, children were classified as SP-3D:AOR or SP-3D:TOR if they scored 2 or above. With these direct assessment cut-off scores, 31% of children with NDD were classified as having AOR and 27% having TOR (Table 5 and Additional file 4: Tables S1–S3 for additional categorical information). Using the SSP parent report, children were classified as SSP:AOR if they scored 5 or above and SSP:TOR if they scored 11 or above. Thus, using parent report, 62% of the children with NDD were classified as having AOR, whereas 68% had TOR. The inter-test agreement between SSP and SP-3D:A for AOR was 65% and TOR was 50%. Based on a two-proportion *z* test for SP-3D:AOR, SSP:AOR, SP-3D:TOR, and SSP:TOR, the NDD group was significantly more affected by SOR than the TDC group (*χ*^2^ ≥ 17.5, *p* ≤ .0001).

**Table 5:** Count and percentage of children with auditory or tactile over-responsivity

	NDD SOR/total (% SOR)	TDC SOR/total (% SOR)
SP-3D:AOR	55/176 (31)	13/127 (10)
SP-3D:TOR	48/176 (27)	9/128 (7)
SSP:AOR	86/138 (62)	11/89 (12)
SSP:TOR	97/143 (68)	8/92 (9)

Percentages reflect percent of children in their respective cohort meeting cut-off criteria for SOR

*SP-3D:A* Sensory Processing-Three Dimensions: Assessment, *SSP* Short Sensory Profile

### Experiment 2

**Fig. 1:**
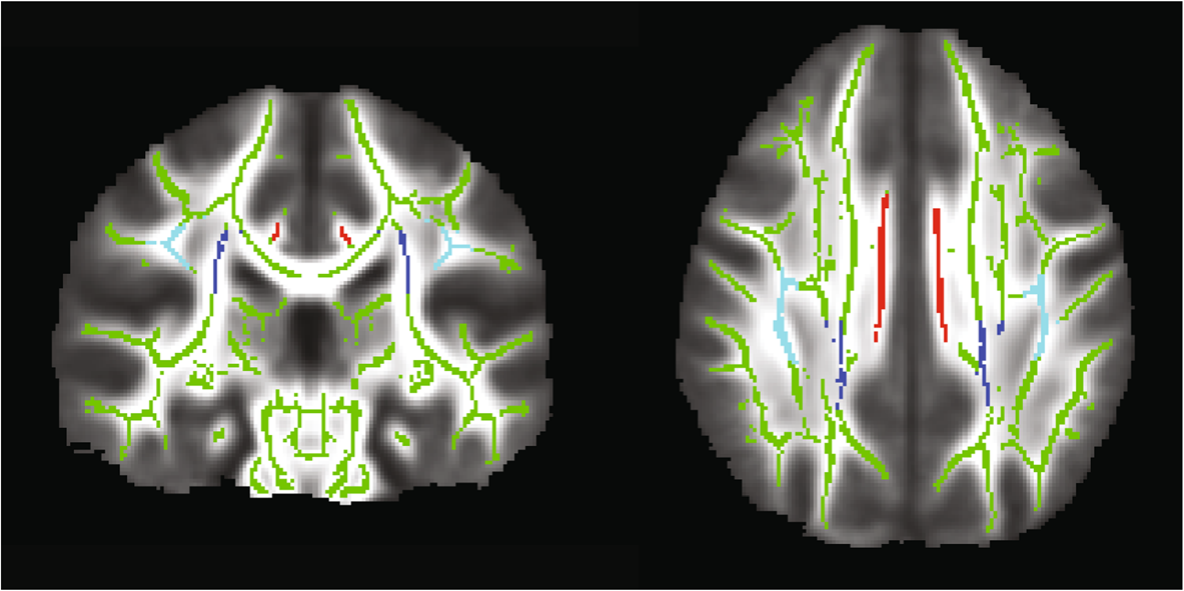
Skeletonized map of FA tracts. Image of the FA skeleton mask (green) displaying the tracts with significantly lower FA in the AOR group: the bilateral posterior corona radiata (PCR, dark blue), superior longitudinal fasciculus (SLF, light blue), and the cingulate gyrus-cingulum portion (CGC, red)

The second aim of our study was to explore the neural mechanisms contributing to AOR based on direct assessment. We compared DTI tracts from children who also completed the SP-3D:A. Based on our SP-3D:AOR cut score analysis, 15 children (3 TDC, 7 ASD, 5 SPD) met AOR threshold and 24 did not. The AOR and NO-AOR cohorts did not differ in age (*p* = .37), perceptual IQ (*p* = .35), or verbal IQ (*p* = .53). We found that children with AOR had 11 total tracts showing decreased FA relative to children without AOR. Given the concern for multiple comparisons with this data-driven approach, we applied FDR correction and three tracts continued to exceed the specified *p* value of < 0.05. These tracts are the posterior corona radiata (PCR), cingulate gyrus-cingulum portion (CGC), and superior longitudinal fasciculus (SLF) ( see Table 6 and Fig. 1).

**Table 6:** DTI tracts showing decreased FA in the auditory over-responsive cohort

Tracts	AOR vs. NO-AOR FA comparisons Uncorrected *p* value (FDR-corrected *p* value)
ICP	.029 (.08)
CP	.023 (.08)
ALIC	.04 (.09)
PTR	.01 (.06)
ATR	.03 (.08)
**PCR**	**.004 (.03)**
EC	.03 (.08)
**CGC**	**.004 (.03)**
**SLF**	**.003 (.03)**
IFOF	.05 (.10)
ILF	.04 (.09)

This table displays group effects on Fractional Anisotropy. *ICP* inferior cerebellar peduncle, *CP* cerebral peduncle, *ALIC* anterior limb of the internal capsule, *PTR* posterior thalamic radiation, *ATR* anterior thalamic radiation, *PCR* posterior corona radiata, *EC* external capsule, *CGC* cingulate gyrus-cingular portion, *SLF* superior longitudinal fasciculus, *IFOF* inferior fronto-occipital fasciculus, *ILF* inferior longitudinal fasciculus. Bolded *p* values indicate statistically significant effects after FDR correction

## Discussion

Sensory processing dysfunctions, specifically sensory over- and under-responsivity, are now part of the DSM-5 criteria for ASD [4]. However, sensory processing challenges are also reported in children with other categorical conditions including ADHD and it is the principle behavioral symptom for children with isolated sensory processing disorder. This growing recognition has motivated the need for better clinical and research measures to characterize sensory processing. Here, in line with the RDoC framework, we investigate SOR in the auditory and tactile domains as a dimension independent of clinical condition. We show that auditory and tactile over-responsivity can be quantified directly for children with and without NDD and that direct assessment has moderate concordance with parent report measures. Second, we report three neural tracts that differentiate children with AOR from those without in a pilot cohort, an exploratory result that needs to be confirmed in larger-scale follow-up studies.

Development of reliable sensory tools, both parent reports and direct assessments, is a critical step for researchers and clinicians alike. We hypothesized that AOR and TOR group assignment utilizing a combined parent report/direct assessment methodology, similar to that used as gold standard diagnosis in ASD, would provide a more reliable sensory cohort assignment and that this combined assessment might be more robust for use with structural neuroimaging analysis. However, we found that the parent report questionnaire and direct observation have only a moderate overlap. Specifically, the agreement between SSP and SP-3D:A for AOR was 65% and TOR was 50%; in other words, 65% of children who met AOR criteria on the parent report also met criteria on direct assessment and 50% of children who met TOR criteria on the parent report also met criteria on the direct assessment. This divergence in parent report versus direct assessment is similar to previous work showing moderate or limited agreement between a sensory questionnaire and direct observation [5, 19]. Tavassoli et al. found an inter-rater agreement between questionnaire and observation of 74%; however, general sensory processing was evaluated rather than auditory and tactile over-responsivity [5]. Schoen et al. focused on SOR and reported a moderate correlation of .47, similar to our findings [19]. In line with previous reports, we find that more children meet SOR criteria based on parent report than on direct assessment in both the auditory and tactile domains, suggesting that the direct assessment may be a more stringent measure. It is worth noting, however, that the rates of auditory and tactile challenge are similar in the NDD group within each measure format. We would expect the TDC group percentiles to be similar and fixed as the grouping method was based on their results. There are several plausible explanations for the higher detection in parent report than direct assessment. First, parent report is subjective due to parental bias and recollection bias. A second explanation for a broader catchment using parent report is that parents have more chances to observe their child’s sensory reactivity symptoms across various environments; thus, a stable trait will be more evident. In a laboratory setting, the amount of sensory stimuli is controlled for and does not represent the vast amount of sensory stimuli a child might experience in everyday situations. Therefore, parent reports likely reflect their child’s atypical behavior across settings to be more abundant than in the laboratory. Finally, it is possible that parents of children with sensory and neurodevelopment differences are more likely to rate their children as affected due to their additional knowledge and concern around aspects of atypical neurodevelopment.

For clinical utility, we suggest using a combination of measures to identify children at risk, such as a sensory questionnaire and clinical assessment. We are not suggesting the use of the cohort assignment from this research sample for clinical determination but rather to assist in understanding of the currently available methods and tools. The goal is to detect all children who might have sensory modulation challenges that interfere with learning and social engagement and to be able to clinically intervene as early as possible. For research purposes, however, we suggest the use of sensory questionnaires as a screening tool, followed by standardized direct observations. Quantitative direct observation measures should be used when investigating biological mechanisms. Future research with larger sample sizes and testing across multiple domains is needed to test these assumptions. Future research should explore the link between sensory questionnaires, observational measures, and psychophysiological measures of sensory perception.

With regard to the best method for revealing brain behavior relationships, a more singular, direct assessment has been shown to correlate better than parent report for sensory discrimination, so it is not surprising that the same could be found for sensory modulation over-responsive subtype [32]. We previously reported wide-spread differences in white matter microstructure in children with SPD and ASD relative to TDC [45]. However, as we have reported in our somatosensory magnetoencephalography work, neural mechanisms can often be better understood by splitting groups not by a clinical label, such as ASD, but by a more narrow construct of interest, such as tactile sensitive versus tactile typical [42]. Engaging a similar approach in this investigation, we split our cohort not by traditional clinical labels (ASD, SPD, or TDC) but by a direct measure of AOR.

We conjectured that a sensory-first phenotype, in this case AOR, allows for a more parsimonious identification of key neural tracts. Indeed, in our previous work based on parent report and a broad inclusion criteria for sensory processing dysfunction, we found decreased FA in children with SPD in the posterior body and isthmus of the corpus callosum, the left posterior thalamic radiations (PTR), left PCR, and the posterior aspect of the left SLF [32]. Here, for children with AOR, the PCR, CGC, and SLF tracts showed decreased FA. In this analysis, the isthmus, posterior body of the corpus callosum, and the PTR were not significantly different between AOR and NO-AOR cohorts. While one might postulate that the current analysis was underpowered to detect the difference, this is unlikely given that the original study had 16 children in the general SPD group and 24 children in the TDC group, which is roughly similar to the 15 AOR and 24 NO-AOR children in this present study. We submit, instead, that the PCR, SLF, and CGC may represent critical connections in an AOR network. Additional work in a larger sample that will allow for investigation of TOR to determine if this network is a shared over-responsivity network or specific to the auditory domain is needed. In addition, a larger sample will allow for comparisons of SOR architecture in children with additional neurodevelopmental domains of challenges such as dysgraphia, dyspraxia, or sustained attention deficits. More broadly, this research adds to a growing body of literature associating the neural contributions of sensory over-responsivity.

### Future directions and limitations

As with any study, there are limitations. First of all, the gender distribution between the neurodevelopmental and TDC group was different given the higher male to female ratio in autism. Moreover, for experiment 1, groups were not matched on cognitive abilities or age. Nevertheless, this should not have affected our analysis; for experiment 1, we do not compare groups but rather use the TDC values for SOR group assignment in the NDD cohort. In experiment 2, the DTI analysis, sex, age, and cognitive abilities were matched. Consequently, investigation in a cohort with both males and females is essential. Second, although over 300 participants took part in our first analysis, only 39 participants took part in the DTI imaging experiment. Consequently, the TOR group with neuroimaging available consisted of only eight children which were insufficient for statistical comparison. For future SOR neuroimaging studies, a larger group of children with mixed neurodevelopmental profiles will allow for a broader range of sensory function. Furthermore, large and broad NDD cohorts will facilitate the understanding of whether SOR differences are fundamentally related to the current categorical cohorts such as ASD or SPD and whether sensory typical children can be included in the continuum for neural mapping. However, emerging genetic findings, imaging reports, and even overlap in clinical semiology for individual children suggest that SOR will not respect these clinical divisions.

Another limitation is that the cingulum bundle was divided into two portions, the superior and hippocampal region. While this is standard convention, reports that suggest a finer parcellation of the CGC into retrosplenial and subgenual divisions to better reflect the independent connections should be considered [56].

Future studies will need to include a larger cohort of individuals with and without neurodevelopmental concerns to better understand other sensory phenotypes, such as sensory under-responsivity (SUR) and sensory seeking. The current study is a first step in understanding the relationship between parent and direct assessment and neural underpinnings of sensory over-responsivity using existing measures. The findings prompt the development of a more integrated parent and direct assessment battery as well as the development of a large normative dataset for standardization. In future studies, we hope to also move beyond group analysis to be able to study sensory over-responsivity as a continuum, which will not only yield important insights into sensory challenges, but also the sensory strengths that have been reported for many individuals with autism such as enhanced visual search and auditory perception [57, 58].

## Conclusions

This study identified cut scores for AOR and TOR using both a parent report measure and direct observation. The SSP parent report and SP-3D:A direct observation ratings overlapped moderately for AOR and TOR. The direct observation measure here, the SP-3D:A, can be used in clinical and research settings to augment SOR phenotyping and further investigate underlying mechanisms of sensory modulation.

## Additional files

Additional file 1: **Figure S1.** Auditory and tactile normative distribution in the TDC cohort. Auditory over-responsivity on the SP-3D:A and SSP. Tactile over-responsivity on the SP-3D:A and SSP (PDF 196 kb)

Additional file 2: **Figure S2.** Auditory and tactile normative distribution in the ASD cohort. Auditory over-responsivity on the SP-3D:A and SSP. Tactile over-responsivity on the SP-3D:A and SSP (PDF 222 kb)

Additional file 3: **Figure S3.** Auditory and tactile normative distribution in the SPD cohort. Auditory over-responsivity on the SP-3D:A and SSP. Tactile over-responsivity on the SP-3D:A and SSP (PDF 219 kb)

Additional file 4: **Table S1.** Percentage of children with auditory or tactile over-responsivity. **Table S2.** Children with over-responsivity at two standard deviations above the mean. Table S3. Sensory over-responsivity scores by categorical group on direct and parent assessment (DOCX 23 kb)

## Abbreviations

ADOS-2: Autism Diagnostic Observation Schedule, Second Edition
AOR: Auditory over-responsivity
AQ: Autism quotient
ASD: Autism spectrum disorder
DSM-5: Diagnostic and Statistical Manual-5
DTI: Diffusion Tensor Imaging
FA: Fractional anisotropy
FDR: False detection rate
ILF: Inferior longitudinal fasciculus
NDD: Neurodevelopmental disorders
RDoC: Research Domain Criteria
ROI: Region of interest
SCQ: Social Communication Questionnaire
SOR: Sensory over-responsivity
SP: Sensory Profile
SP-3D:A: Sensory Processing-Three Dimensions: Assessment
SPD: Sensory processing dysfunction
SSP: Short Sensory Profile
TDC: Typically developing children
TOR: Tactile over-responsivity
