# Supplementary material for: Sensory over-responsivity: parent report, direct assessment measures, and neural architecture
Source: Mol Autism. 2019 Feb 4;10:4. doi: 10.1186/s13229-019-0255-7 (PMC6360663; doi:10.1186/s13229-019-0255-7)
Supplement: Supplementary file 2 — Figure S2. Auditory and tactile normative distribution in the ASD cohort. Auditory over-responsivity on the SP-3D:A and SSP. Tactile over-responsivity on the SP-3D:A and SSP (PDF 222 kb) [file 13229_2019_255_MOESM2_ESM.pdf]

2a.

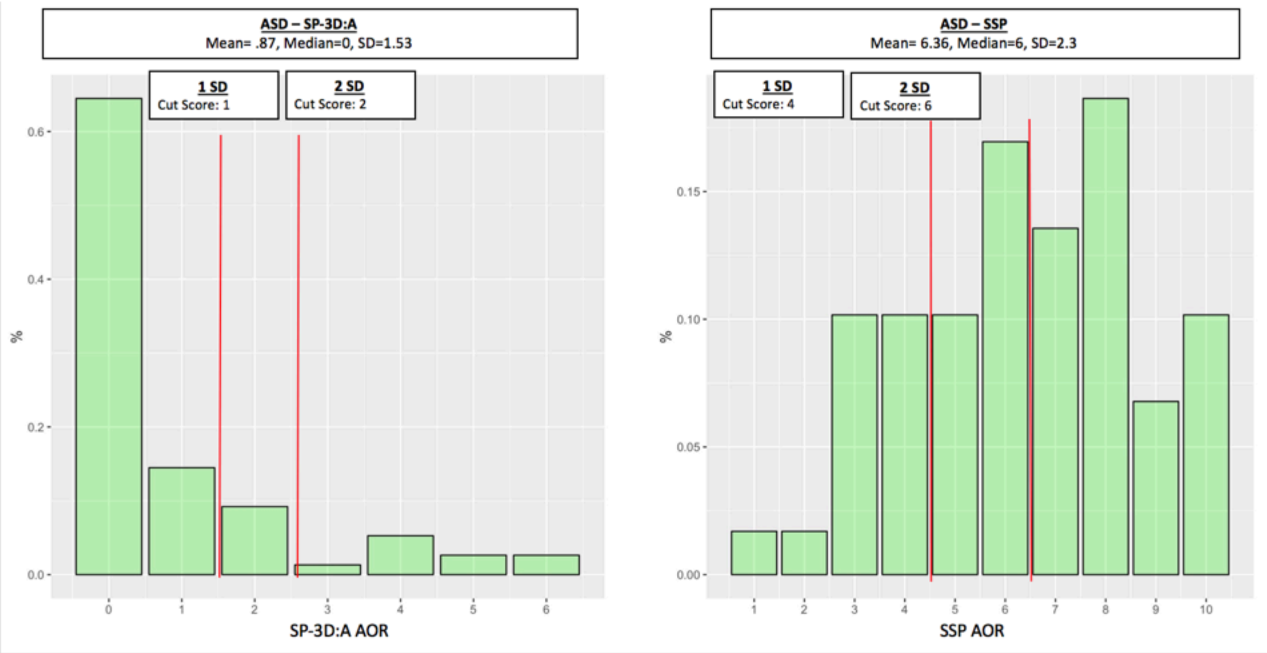

ASD= Autism Spectrum Disorder; SP-3D:A= Sensory Processing-3 Dimensions: Assessment, SD= Standard Deviation; AOR= Auditory Over-Responsivity; SSP= Short Sensory Profile

2b.

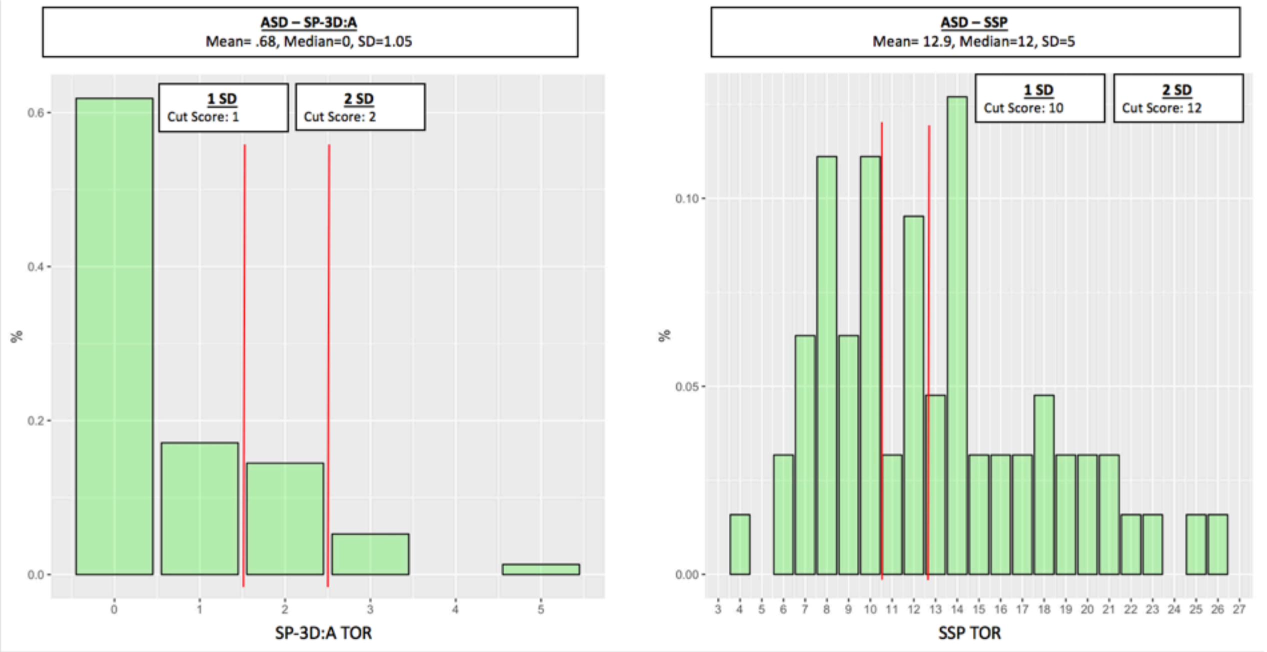

ASD= Autism Spectrum Disorder; SP-3D:A= Sensory Processing-3 Dimensions: Assessment, SD= Standard Deviation; TOR= Tactile Over-Responsivity; SSP= Short Sensory Profile
