# Supplementary material for: Sensory over-responsivity: parent report, direct assessment measures, and neural architecture
Source: Mol Autism. 2019 Feb 4;10:4. doi: 10.1186/s13229-019-0255-7 (PMC6360663; doi:10.1186/s13229-019-0255-7)
Supplement: Supplementary file 4 — Table S1. Percentage of children with auditory or tactile over-responsivity. Table S2. Children with over-responsivity at two standard deviations above the mean. Table S3. Sensory over-responsivity scores by categorical group on direct and parent assessment (DOCX 23 kb) [file 13229_2019_255_MOESM4_ESM.docx]

**Additional file 4: Table S1. Percentage of Children with Auditory or Tactile Over-Responsivity**

|  | ASD  % SOR | SPD  % SOR |
| --- | --- | --- |
| SP3D:AOR | 21% | 39% |
| SP3D:TOR | 21% | 32% |
| SSP:AOR | 76% | 52% |
| SSP:TOR | 60% | 74% |

*Percentages reflect percent of children in their respective cohort meeting cut-off criteria for SOR.*

*SP-3D:A = Sensory Processing- Three Dimensions: Assessment*; *SSP = Short Sensory Profile Parent Report*

**Table S2. Children with Over-responsivity at 2 Standard Deviations Above the Mean**

|  | TDC  % SOR (+2 SD) | NDD  % SOR  (+2 SD) |
| --- | --- | --- |
| SP3D:AOR | 5% | 15% |
| SP3D:TOR | 2% | 12% |
| SSP:AOR | 2% | 36% |
| SSP:TOR | 4% | 53% |

*Percentages reflect percent of children in their respective cohort with SOR based on a >2 Standard Deviations above the mean cut-off.*

*SP-3D:A = Sensory Processing- Three Dimensions: Assessment*; *SSP = Short Sensory Profile Parent Report*

**Table S3. Sensory Over-Responsivity Scores by Categorical Group on Direct and Parent Assessment**

|  | TD Mean ± SD,  range (n) | ASD Mean  ± SD, range (n) | SPD Mean  ± SD, range (n) |
| --- | --- | --- | --- |
| *SP-3D:A* - Auditory | .38 ± .94,  0-6 (127) | .87 ± 1.5,  0-6 (76) | 1.3 ± 1.4,  0-6 (100) |
| *SP-3D:A* - Tactile | .29 ± .72,  0-5 (128) | .68 ± 1.0,  0-5 (76) | 1.1 ± 1.,2  0-5 (100) |
| SSP - Auditory | 3.0 ± 1.4,  2-7 (89) | 6.3 ± 2.3,  2-10 (59) | 5.1 ± 2.3,  2-10 (79) |
| SSP - Tactile | 7.5 ± 2.2,  6-16 (92) | 13.0 ± 5.0,  6-26 (63) | 14.0 ± 4.9,  6-27 (80) |
